# Combined hepatectomy and radiofrequency ablation for multifocal hepatocellular carcinoma: a case report

**DOI:** 10.4076/1757-1626-2-7987

**Published:** 2009-06-19

**Authors:** Luigi Sandonato, Calogero Cipolla, Maurizio Soresi, Giuseppe Lo Re, Federica Latteri, Giuseppina Lombardo, Valentina Bova, Mario Adelfio Latteri

**Affiliations:** 1 Department of Oncology, Division of General and Oncological Surgery, University of Palermo, Interdepartmental Unit for Hepatic Neoplasia GroupVia del Vespro 129, 90127 PalermoItaly; 2 Department of Clinical Medicine and Emerging Pathologies, Division of Internal Medicine and Hepatology, University of Palermo, Interdepartmental Unit for Hepatic Neoplasia GroupVia del Vespro 129, 90127 PalermoItaly; 3 Department of Medical Biotechnology and Legal Medicine, Division of Radiological Science, University of Palermo, Interdepartmental Unit for Hepatic Neoplasia GroupVia del Vespro 129, 90127 PalermoItaly; 4 Biomedical Department of Internal and Specialised Medicine, Division of Gastroenterology and Hepatology, University of Palermo, Interdepartmental Unit for Hepatic Neoplasia GroupVia del Vespro 129, 90127 PalermoItaly

## Abstract

**Introduction:**

At the present time, the best possible choice for the local management of a multifocal hepatocellular carcinoma (HCC) developing on liver cirrhosis is multimodal treatment of the disease. Combined approach based on simultaneous radiofrequency ablation (RFA) together with limited surgical resection represents a valid choice of treatment.

**Case presentation:**

A 75-year-old white female patient affected of HCV-associated cirrhosis in Child-Pugh’s functional class A5, developed a bifocal HCC. The patient had undergone a limited surgical resection together with simultaneous RFA, without intraoperative and postoperative surgical complications. At 36 months after surgery, still shows no sign of disease relapse.

**Conclusion:**

This strategy directed at the management of multifocal HCC, may prove more useful for the reduction of surgical risk and post-operative progression of the liver cirrhosis than large-scale hepatectomy, since it presents no peri-operative mortality and a complication rate of less than 10%.

## Introduction

The treatment of hepatocellular carcinoma (HCC) developing on liver cirrhosis is conditioned by various factors, which are linked both to the cirrhosis and to the tumour. The site, together with the number of lesions and their sizes, may considerably reduce the possibility of choosing a really effective therapeutic management [1].

One of the most important difficulties at the time of diagnosis regards multifocality, since even in the case of candidates for surgical treatment, the site of the lesion may involve the sacrifice of a large area of parenchyma which may not be compatible with the underlying cirrhotic tissue. Furthermore, in an attempt to conserve the hepatic parenchyma and in those cases where surgery is not indicated, loco-regional procedures have been widely used, for example, radiofrequency ablation (RFA), which offers a low rate of complications and considerable therapeutic efficiency, especially with regard to lesions of less than 3 cm [2].

On the basis of these considerations, therefore, in the last few years a multimodal approach has been proposed for the management of multifocal HCC, with different timing and various procedures depending on the specific presentation of the disease. In cases of patients with compensated cirrhosis who are candidates for surgical management, one possibility is combined hepatectomy and RFA, performed during the same procedure, which makes it possible to remove the most peripheral lesion and also to treat any other lesions which, depending on their site, may otherwise have required large-scale removal of hepatic parenchyma [3].

## Case presentation

**Figure 1.**
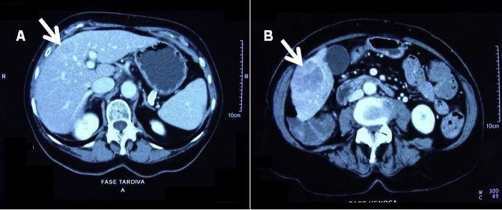
(**A**) Focal lesion on S4. (**B**) Focal lesion on S6.

A 75-year-old white female patient treated by us for about 15 years for HCV-associated cirrhosis in Child-Pugh’s functional class A5. The ultrasound examination showed two focal lesions, one of 2.1 cm in diameter on the IV hepatic segment (S4) and the other of 4.0 cm in diameter on the VI hepatic segment (S6), both of which hypoechogenic and related to an HCC. An abdominal CT with contrast medium confirmed the presence of the two lesions, with a typical HCC contrastographic result, showing a marked enhancement in the arterial phase and wash-out in the portal phase, which was delayed and presented a persistently dense peripheral margin (Figure 1). No increase in serum levels of alpha pheto-protein, which was 35 ng/ml, was observed during follow-up.

The functional class of the cirrhosis, together with the site and size of the lesions, suggested the use of a simultaneous, combined approach with RFA of the S4 lesion and resection of the S6 lesion.

**Figure 2.**
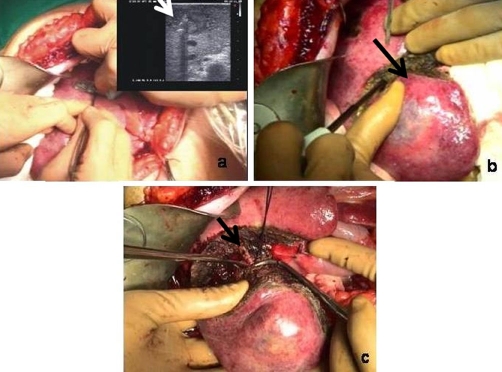
(**A**) Intraoperative ultrasound and RFA of the S4 lesion. (**B**) Segmentectomy of the S6 lesion. (**C**) selective ligature of the segmentary peduncle.

After partial mobilisation of the right hepatic lobe, intraoperative ultrasound was performed in order to exclude the presence of any further lesions, followed by RFA of the S4 lesion with a Model 1500 generator (RITA Medical System, Mountain View, CA), using an ultrasound-guided needle-electrode provided with expandable hooks which were gradually opened during the 21 minutes of the procedure (Figure 2a).

As a precaution, an elastic ligature was then applied to the hepatic peduncle and subsequently, without clamping, segmentectomy was performed for the removal of the S6 lesion (Figure 2b). The resection required the use of a TissueLink FB 3.0 Floating Ball radiofrequency handpiece (RITA Medical System, Mountain View, CA) and terminated with the selective ligature of the segmentary peduncle (figure 2c), identified by means of ultrasound. Blood loss from the resected surface was less than 50 cc. Finally, a subhepatic drainage tube was put into position.

The post-operative period was normal, without any particular changes in the RBC or HGB count; the preoperative values of these were, respectively, of 5.150 million/ml and 13.8 gm/dl, with an Ht of 42.6, while the immediate postoperative values were RCB 4,900 million/ml, HGB 13.4 gm/dl and Ht of 40.9. There was only a slight increase in post-operative transaminases, which reached 459 for GOT and 158 for GPT on post-operative day 1, returning to normal on day 5. We also observed an increase in total bilirubinemia, which on day 1 went from 0.89 to 2.19, with a clear prevalence in the indirect quota; these values returned to normal on day 4.

The drainage was removed on day 2, when the patient resumed oral feeding; she was sent home on day 6.

The biopsy of the resected lesion confirmed the diagnosis of encapsulated HCC with a mainly pseudoglandular growth pattern, Edmondson’s grade II; the resection margins proved to be healthy.

The patient has been followed up every six months and now, at 36 months after surgery, still shows no sign of disease relapse.

## Discussion

Nowadays, careful screening protocols permit the diagnosis of HCC in an earlier and earlier phase of the disease, with the resulting possibility of efficient therapeutic management in more and more cases [4]. The size of the lesion at diagnosis is an extremely important factor, since those of less than 3 cm treated with RFA present a local relapse rate of less than 10% [2], which means that the use of this procedure is becoming more and more frequent, especially in those patients where surgical management is not indicated.

Nowadays, liver surgery is considered to be the best approach to early-stage HCCs in patients with cirrhosis who are not candidates for liver transplant [5], and presents the lowest local relapse rate. Nevertheless, in such cases, depending on the site of the neoplasia, and where small tumours are concerned, RFA should be considered as a valid alternative to surgical resection [6], which should be reserved for use in cases of disease relapse in other segments, since this is part of the natural history of HCC and may occur after various periods of time in over 70% of cases.

One of the most complicated problems involved in the difficult management of HCCs is the presence of a multifocal neoplasia, which may not permit the use of one procedure only, but may require a multimodal approach, all at the same or at different periods, so that it may not be possible to consider the therapeutic value of each single procedure individually.

In this context also, resective treatment is still extremely important, possible associated with efficient loco-regional procedures, both at one and the same time (RFA) [2,3] and with different timing (TACE).

With regard to surgical management, it should be borne in mind that the last few years have seen the development of more and more sophisticated techniques and the use of more and more efficient instruments, making it possible to perform several procedures presenting a lower mortality rate and fewer and fewer complications, and, moreover, without the need to use clampage of the hepatic peduncle; this means that such procedures can be used in patients with low grade portal hypertension, with an extremely limited blood loss and a much shorter operating time [7]. A further apparent advantage deriving from the use of RFA is the possibility of “sterilising” the resected surface, thus producing an area of coagulative necrosis of about one cm and reducing the risk of local relapse. In most cases, RFA can be performed subcutaneously and may be effectively associated with surgical procedures, especially in smaller lesions (< 3 cm) whose site would otherwise involve the large-scale removal of hepatic parenchyma in patients where the disease will inevitably bring about liver failure [3].

Therapeutic management consisting of hepatic resection combined with simultaneous RFA, initially proposed for the treatment of patients with colorectal liver metastases [8], is, in our opinion, particularly indicated for patients with larger peripheral tumours, where a limited resection is possible, together with one or two tumoral nodules of less than 3 cm.

In conclusion, we maintain that this strategy directed at the management of multifocal HCC, may prove more useful for the reduction of surgical risk and post-operative progression of the liver cirrhosis than large-scale hepatectomy, since it presents no peri-operative mortality and a complication rate of less than 10% [3].

## List of abbreviations

HCC: hepatocellular carcinoma
RFA: radiofrequency ablation
S4: IV hepatic segment
S6: VI hepatic segment
TACE: trans arteriosous chemoembolizzation
